# OculoMotor & Vestibular Endurance Screening (MoVES) Normative, Repeatability, and Reliability Data

**DOI:** 10.3390/brainsci14070704

**Published:** 2024-07-14

**Authors:** Stephanie Iring-Sanchez, Michaela E. Dungan, Andrew Jones, Mitchell Malakhov, Stuti Mohan, Chang Yaramothu

**Affiliations:** 1 Massachusetts Eye and Ear, Department of Otolaryngology-Head and Neck Surgery, Harvard Medical School, Boston, MA 02114, USA; siring-sanchez@meei.harvard.edu; 2 School of Applied Engineering and Technology, New Jersey Institute of Technology, Newark, NJ 07102, USA; med38@njit.edu (M.E.D.); abj7@njit.edu (A.J.); mm2483@njit.edu (M.M.); sm57@njit.edu (S.M.)

**Keywords:** concussions, traumatic brain injury, head injury, endurance, injury recovery, eye movements, vestibular dysfunction

## Abstract

This study aims to assess oculomotor and vestibular endurance by utilizing the Oculomotor and Vestibular Endurance Screening (MoVES) assessment in athletes’ pre-season and post-season and after a suspected head injury to detect impairment. Athletes (*N* = 311, 19.4 ± 1.3 years) were recruited to perform the following seven tasks: (1) horizontal saccades, (2) vertical saccades, (3) vergence jumps, (4) horizontal vestibular-oculomotor reflex (VOR), (5) vertical VOR, (6) amplitude of accommodation (AoA), and (7) near point of convergence (NPC). At pre-season, the observed number of eye movements in 60 s are horizontal saccades (74 ± 13 initial 30 s; 67 ± 11 latter 30 s), vertical saccades (70 ± 13; 66 ± 10), vergence jumps (48 ± 12; 45 ± 13), horizontal VOR (38 ± 11; 38 ± 11), and vertical VOR (8 ± 11; 38 ± 11). These results establish a normative database for eye movements within the MoVES assessment and show consistency in the number of movements from pre-season to post-season. The initial results show a trending decrease in the number of eye movements in the initial days post-head injury, which improves to pre-season measures 14–21 days post-injury. This foundation can be used by future studies to explore the extent of binocular and vestibular endurance dysfunctions caused by head injuries that subside within two weeks.

## 1. Introduction

There are over 3.8 million sports-related concussions annually in the United States alone, making concussions a major health problem. According to the Sixth International Conference on Concussion in Sport, a concussion is defined as a direct blow to the head, neck, or body that results in the transmission of force to the brain which occurs during exercise-related activities or sports [1]. Visual symptoms, such as difficulty reading, blurred vision, difficulty focusing, and eye fatigue, are commonly reported following a concussion [2,3]. Vision disorders are common in patients with persistent symptoms following a concussion, with studies reporting that 69% have a minimum of one associated vision disorder [3] and 62.5% have vestibulo-ocular dysfunction [4]. The fascial network plays an important role in the function and mobility of the cranial and extraocular muscles [5,6,7]. Concussion-induced alterations in this network can profoundly impact eye movements, leading to several visual disorders. Therefore, an instrument that can capture visual and vestibular impairment has a high probability of detecting and monitoring potential head injuries and concussions. This study seeks to establish normative, repeatability, and reliability data on the OculoMotor & Vestibular Endurance Screening (MoVES) assessment, which may be used as an objective measure to enhance current post-concussion tests.

Concussion-related vision disorders may include (1) convergence insufficiency, the inability to converge, which often leads to problems with reading, eye fatigue, and eye strain [3,8,9,10,11,12]; (2) accommodative dysfunction, the inability to change focus from a distance to a near target, often leading to blurred vision with near tasks, headaches, and fatigue [3,13]; and/or (3) eye movement dysfunction involving saccades, which is the ability to rapidly move from one target to another (vertically or horizontally) and is used for most visual tasks, such as reading and athletic coordination [3,8,14]. For this reason, “return to learn” and “return to sport” testing batteries should include more than just symptoms, cognitive function, and balance; they should also include oculomotor and vestibular function screenings [1]. 

Concussion screening tools measure the severity of symptoms following limited tasks (i.e., vestibular/ocular motor screening (VOMS)) [8,15]. The VOMS for concussion includes smooth pursuits, horizontal and vertical saccades, the measurement of near point of convergence, horizontal and vertical vestibular-ocular reflex, and visual motion sensitivity [8]. This test requires the examiner to hold their finger up as the target at 3 ft away from the participant and move it 1.5 ft (left or right/up and down) so that the participant follows the target (10 repetitions) and then reports any symptoms including headache, dizziness, nausea, and fogginess on a scale of 0–10. Factors that could potentially limit results include (1) a participant with mild symptoms may not sense any symptoms following the initial oculomotor task, which may lead to false negatives, (2) the subjective measure of the test may also lead to false negatives and false positives, (3) although the targets are cost-effective, they may vary from examiner to examiner or time point to time point, and (4) the limited number of repetitions (10) may not evoke any symptoms, leading to false negatives [16,17,18]. Additionally, while vergence and accommodative dysfunction are commonly reported post-concussion, the VOMS assessment does not include a vergence jumps (facility) test, only a static near point of convergence measure. Additionally, it does not include an assessment for accommodative amplitude [3,8,9,10,11,12]. Therefore, the addition of objective metrics, oculomotor endurance (through the inclusion of more repetitions) with fixed targets to facilitate repeatability of the assessment, and a more detailed assessment of vergence jumps may aid clinicians in identifying oculomotor dysfunction by increasing the sensitivity and specificity of post-concussion examinations [19]. Endurance is defined as the ability to sustain a repetitive activity. 

The purpose of this manuscript was to collect and provide normative data on the MoVES assessment in Division I (highest level of intercollegiate athletics in the United States) athletes at pre-season and post-season time points. As a pilot feasibility study, data were collected on a subset of players who experienced a suspected head injury to examine the tool’s predictive ability in identifying oculomotor and vestibular abnormalities post-head injury. We hypothesized that (1) there would be trending decreases in the number of observed eye movements and a receded near point of convergence and accommodation immediately post-head injury that resolves within one month post-head injury, and (2) there would be decreases in the number of eye or head movements, near point of convergence, or amplitude of accommodation that persist beyond four weeks, which may indicate persistent oculomotor and/or vestibular dysfunctions. 

## 2. Materials and Methods

### 2.1. Participants

A total of 311 NCAA Division I level athletes (31% assigned female and 69% assigned male at birth) were recruited from the New Jersey Institute of Technology in Newark (NJIT), NJ. Participants aged 18–24 years were eligible to participate in this study. Exclusion criteria included loss of vision and amblyopia. Pre-season data were collected on athletes within the men’s baseball, men’s and women’s basketball, men’s and women’s cross country, men’s and women’s fencing, men’s lacrosse, men’s and women’s soccer, men’s swimming and diving, men’s and women’s tennis, men’s and women’s track and field, and men’s and women’s volleyball teams. If an athlete experienced a suspected concussion or self-reported a head injury to an athletic trainer during a game or practice, they were referred to the study team. Data points were collected on the athletes immediately after their suspected concussion within the first 24 h, if possible, and 2, 4, 7, 14, 21, 30, and 60 days following the head injury. 

### 2.2. Equity, Diversity, and Inclusion Statement

This study was open to all NCAA Division I student-athletes at NJIT in Newark, NJ, and was inclusive of all sexes, genders, races/ethnicities, socioeconomic levels, and occurrences in a marginalized community. The research team included 3 women and 3 men (biomedical engineering and biomedical sciences). One of the authors is Hispanic, and four of the authors are junior scholars. Our analysis includes sex as a covariate; however, we acknowledge we did not assess the effects of race/ethnicity or socioeconomic status. 

### 2.3. Data Collection and Protocol

All procedures were approved by NJIT’s Institutional Review Board in accordance with the Declaration of Helsinki. Informed written consent was obtained prior to data collection. Data were collected in the NJIT athletic facilities. Information, including demographics, previous concussion history (diagnosed and self-reported), mechanism of injury, and symptomology, was collected. After the demographics were collected, the OculoMotor & Vestibular Endurance Screening (MoVES) assessment was administered. In this study, the term “suspected head injury” is used to define a direct hit/blow to the head during physical activity that resulted in an athlete being sent for an evaluation by athletic training staff. Athletes were classified as normative controls if they did not experience a direct hit/blow to the head in the entire season. The time of injury was self-reported by the athlete or substantiated using a game video.

MoVES was administered to athletes pre-season, post-season, and/or post-head injury. MoVES is a targeted vision and vestibular examination that utilizes clinically available tools, the OculoMotor Assessment Tool (OMAT) and the OMAT smartphone application, to provide objective and quantifiable outcomes that may improve patient-focused interventions and monitor recovery post-concussion/post-head injury [19]. A previous study has previously established normative data on the following eye movement tasks: (1) horizontal saccades, (2) vertical saccades, and (3) vergence jumps [19]. Any measurements outside the normative range may indicate poor endurance and may be used to determine visual or vestibular abnormalities to refer patients to appropriate healthcare professionals [19]. The MoVES assessment includes five oculomotor tests and two vestibular/oculomotor reflex (VOR) tests. The five timed tests record the number of eye movements or head rotations and include (1) horizontal and (2) vertical saccades to measure a person’s ability to rapidly move their eyes in the horizontal and vertical planes (conjugate eye movements), (3) vergence jump eye movements to measure a person’s ability to rapidly converge (cross) and diverge (uncross) when fixating their eyes at a far and near target (disconjugate eye movements), and (4) horizontal and (5) vertical vestibular-oculomotor reflex (VOR) to measure vestibular function by fixating their eye on a target as they rotate their head horizontally and vertically. The test also includes near point of convergence to measure a person’s ability to converge their eyes as a set of target letters approaches them, and amplitude of accommodation to measure optical power in each eye. Following each assessment, participants were asked to report their symptoms “based on how they feel right now” regarding headache, dizziness, nausea, and fogginess, each scaled 0–10 (0 for no symptoms to 10 for feeling severe symptoms). All tests were performed with participants wearing corrective refraction glasses or contacts, if applicable. Detailed instructions for the test are provided in Supplementary S1 and can be found at https://research.njit.edu/sqrl/moves (accessed on 28 May 2024).

This study utilized the OMAT for visual targets and the companion OMAT smartphone application for counting using methods established by Yaramothu et al. [19]. The examiner selected horizontal, vertical, or jumps as an option in the OMAT smartphone application and then pressed the “START” or “COUNT” button to begin counting the number of eye movements for each of the five timed tests. The examiner pressed the count button for every eye movement. For example, left to right (horizontal) is one movement, top to bottom (vertical) is one movement, and far to near is one movement.

The OMAT tool has the following four components: (1) vergence bar, (2) saccade bar, (3) large slider, and (4) small slider. The vergence and saccade bars have a length of 25 cm, the vergence bar has a magnetic and non-magnetic end, and the saccade bar has a magnet in the middle of the bar. The non-magnetic end of the vergence bar was placed on the participant’s nasion for (1) horizontal saccades, (2) vertical saccades, and (3) vergence jumps, which creates a test distance of 25 cm. The larger slider has a solid vertical line (1 × 0.09 cm) on one side and vertical letters on the other [19]. For both the horizontal and vertical saccade tests, the magnetic end of the vergence bar was attached to the middle of the saccade bar. An “X” target (width of 0.13 cm, 0.5 cm in length and height) was printed on a green/red shaded square at each end of the saccade bar. The distance between the center of one “X” to the other was 24.5 cm. Participants were asked to look between the two “X” targets, allowing them to make eye movements approximately 50 degrees in magnitude vertically and horizontally. For vergence jumps, the large slider with a solid line facing the participant was placed 24 cm (Location A) from the nasion for far vergence demand, and a smaller slider with a solid line facing the participant was 8.9 cm (Location F) from the nasion for near vergence demand (the distance between the targets was 15.1 cm for a 20-degree binocular movement). Participants were asked to rapidly change their fixation from the far (larger slider) to the near (small slider) target. The examiner counted the number of eye movements for vertical saccades, horizontal saccades, and vergence eye movements for one minute.

For the horizontal VOR test, participants were asked to completely extend their non-dominant hand, holding the large slider on the side with the letters toward them (at eye level) while they rotated their heads horizontally in the left and right directions as fast as they could while maintaining fixation on the letters with both eyes. For the vertical VOR test, participants were asked to move their heads up and down, fully extending their heads, while holding the large slider with the letters facing them with their dominant hand. The examiner counted each of the horizontal and vertical head rotations for one minute. Left to right was one eye movement, and right to left was another eye movement. A demonstration of horizontal and vertical VOR movements made by the participants is included in Supplementary Video S1.

For near point of convergence, the examiner inserted the large slider into the vergence bar and placed it at the 25 cm marking, with the letters facing the participant. The examiner placed the 1 cm end of the bar on the patient’s nasion and slowly (~ 2 cm/second) moved the slider toward the participant while the participant attempted to maintain fixation on the letters by ensuring they were single. When the participants reported doubling, they were asked to fuse the letters, making them single again. If they could fuse the letters to be single, the examiner moved the slider closer to the participant’s eye and recorded the cm value (to the nearest ½ centimeter) at which the patient reported sustained double vision (breakpoint). The participant was instructed to ignore the target blurring and that double vision was the endpoint. When the participant reported they could not fuse the letters, the examiner moved the slider away from the participant and recorded the cm value at which the participant perceived the letters as single again (recovery point). 

For accommodative amplitude, the examiner placed the 1 cm end of the vergence bar above the right eyebrow. If the participant was wearing glasses, the examiner ensured the participant viewed the target through the glasses. The patient was instructed to cover their left eye with their left hand and maintain focus on the letters, ensuring they were clear. The participants were asked to report when the letters became blurry. The examiner slowly (~2 cm/s) moved the slider toward the participant. The examiner recorded the cm value (to the nearest ½ centimeter) at which the participant reported blurriness. The test was repeated for the left eye. 

### 2.4. Statistical Analysis 

All statistical analyses were conducted using IBM SPSS Version 20. Summary statistics were assessed for continuous variables, including means, standard deviations, minima, maxima, interquartile ranges, and 95% confidence intervals. An initial independent samples *t*-test was conducted to evaluate if there are any significant differences between pre-season data without any post-data (*n* = 191) and pre-season data with post-data (*n* = 120). A two-way repeated measures ANOVA (rmANOVA), with sex and age as covariates, was applied to assess endurance and compare the number of eye movements performed within the initial and latter 30 s at pre-season and post-season for all five timed assessments. Post hoc pairwise comparisons, with α = 0.05/4 = 0.0125, were used to assess the difference in the number of eye movements. A paired samples *t*-test was conducted (within subjects) to assess the amplitude of accommodation (AoA) and near point of convergence (NPC) break and recovery in athletes at pre-season and post-season. 

A 2-way mixed-effect intraclass correlation coefficient was calculated to examine the reliability of pre/post-season data that were collected. ICC values below 0.5 were considered poor reliability, 0.05 to 0.75 was moderate, values between 0.75 and 0.9 were good, and values above 0.9 were considered to demonstrate excellent reliability. A linear mixed model was utilized to identify statistical differences post-head injury at multiple time points. 

## 3. Results

### 3.1. Demographic Characteristics of Athletes and Symptomology

Of the 311 participants recruited for this study, pre-season data were collected on all the participants, and post-season data were collected on 120 of those participants. Post-head injury data were collected from 15 participants. Of the 135 participants with pre-season and post-season data and/or post-head injury data, 93 were men (69%) and 42 were women (31%). The average age of the entire study population was 19.4 ± 1.3 years. The average total number of symptoms (sum of all the symptom values reported after each assessment) reported at pre-season was 5 ± 12 compared to 46 ± 62 immediately post-head injury. Sex was not a significant covariate in any of the assessments; *p* > 0.05. 

### 3.2. Summary Statistics for all Seven Assessments in Athletes at Pre-Season and Post-Season 

Table 1 shows summary statistics in the normative cohort for all seven assessments. Table 2 shows athletes’ pre-season and post-season data. Values presented include the time interval, average (1 standard deviation [±SD]), non-zero minimum, maximum, and median (95% confidence interval). 

An independent samples *t*-test confirmed no significant differences (*p* > 0.05) in the initial 30 s and latter 30 s between players with both pre-season and post-season data (*n* = 120) and players with just pre-season data (*n* = 191).

**Figure 1:**
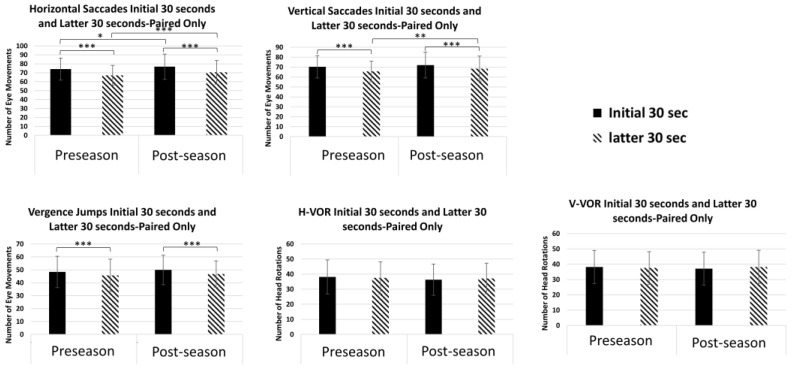
Average for the 5 timed assessments in players at pre-season and post-season. Statistically significant pairs are shown with the following symbols: *p* = 0.005 *, *p* = 0.001 **, *p* < 0.001 ***.

**Figure 2:**
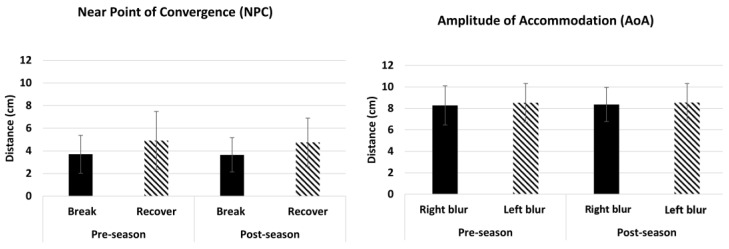
Average of the 2 non-timed assessments (amplitude of accommodation [AoA] and near point of convergence [NPC]) in players at pre-season and post-season.

Figure 1 shows bar plots illustrating the average initial and latter outcomes in each of the five timed assessments. Figure 2 shows bar plots showing the average NPC and AoA outcomes in each of the two assessments.

### 3.3. Performance for Horizontal Saccades 

A two-way repeated measures ANOVA (rmANOVA) confirmed a significant difference in the endurance main effect for horizontal saccades between the initial and latter 30 s, F(1, 116) = 67.10, *p* < 0.001, η^2^ = 0.366; however, there was no statistical difference in the pre-season and post-season main effect, F(1, 116) = 1.17, *p* = 0.283, η^2^ = 0.010. The interaction effect between endurance and pre-/post-season was also insignificant, F(1, 116) = 0.31, *p* = 0.581, η^2^ = 0.003. Age and sex were not shown to be statistically significant for any of the main effects or interactions. Post hoc pairwise comparisons revealed a significant decrease of 7.0 eye movements between the initial 30 s compared to the latter 30 s at pre-season and a significant decrease of 6.3 movements between the initial 30 s compared to the latter 30 s post-season; *p* < 0.001. The ICC showed good reliability in the initial (0.819) and latter (0.793) measurements taken at pre- and post-season. 

### 3.4. Performance for Vertical Saccades

The two-way rmANOVA also showed a significant difference in the endurance main effect for vertical saccades between the initial and latter 30 s, F(1, 116) = 67.08, *p* < 0.001, η^2^ = 0.366; however, it showed no significance for the pre-/post-season main effect [F(1, 116) = 1.55, *p* = 0.215, η^2^ = 0.013] or the interaction effect [F(1, 116) = 1.33, *p* = 0.251, η^2^ = 0.011]. Age and sex were also not statistically significant. Post hoc pairwise comparisons revealed a significant decrease of 4.7 eye movements between the initial 30 s compared to the latter 30 s at pre-season and a significant decrease of 3.6 between the initial 30 s compared to the latter 30 s post-season; *p* < 0.001. The reliability of the vertical saccades can also be classified as good based on the initial (0.783) and latter (0.799) measurements. 

### 3.5. Performance for Vergence Jumps

A two-way rmANOVA showed significance in the endurance main effect [F(1, 116) = 9.286, *p* = 0.003, η^2^ = 0.074] and the interaction effect [F(1, 116) = 9.724, *p* = 0.002, η^2^ = 0.077], but not in the pre/post-season main effect [F(1, 116) = 3.747, *p* = 0.055, η^2^ = 0.031]. Age and sex were not statistically significant. A post hoc pairwise analysis revealed a significant decrease of 2.7 eye movements between the initial 30 s at pre-season compared to the latter 30 s at pre-season and a significant decrease of 3.2 between the initial 30 s compared to the latter 30 s post-season; *p* < 0.001. There were no significant differences between the initial 30 s at pre-season compared to the initial 30 s post-season (*p* = 0.187) or between the latter 30 s at pre-season compared to the latter 30 s post-season (*p* = 0.40). The reliability of the vergence jump measurements can be classified as moderate when comparing pre-/post-season at initial (0.568) and latter (0.546) 30 s.

### 3.6. Performance for Vertical and Horizontal VOR 

A two-way repeated measures ANOVA confirmed there were no significant differences in the endurance [F(1, 116) = 1.288, *p* = 0.259, η^2^ = 0.011] or pre-/post-season [F(1, 116) = 1.866, *p* = 0.175, η^2^ = 0.016] main effects or interaction [F(1, 116) = 2.184, *p* = 0.142, η^2^ = 0.018] for H-VOR. There were also no significant differences in V-VOR between endurance [F(1, 116) = 0.308, *p* = 0.580, η^2^ = 0.003] or pre-/post-season [F(1, 116) = 0.687, *p* = 0.409, η^2^ = 0.006] main effects and interaction [F(1, 116) = 0.030, *p* = 0.862, η^2^ < 0.001] effects. Sex and age remain insignificant in these comparisons. H-VOR had poor reliability in the initial (0.482) and latter (0.45) 30 s; however, V-VOR had poor reliability in the initial (0.378) 30 s but moderate reliability in the latter (0.634) 30 s.

### 3.7. Performance for Amplitude of Accommodation (AoA) and Near Point of Convergence (NPC)

A paired *t*-test demonstrated there were no significant differences in NPC at pre-season versus post-season (NPC break, *p* = 0.76 and NPC recovery, *p* = 0.51). There were also no significant differences in AoA at pre-season compared to post-season (right eye, *p* = 0.66 and left eye, *p* = 0.87).

### 3.8. Summary Statistics in Athletes at Pre-Season, Post-Head Injury and Post-Season

**Figure 3:**
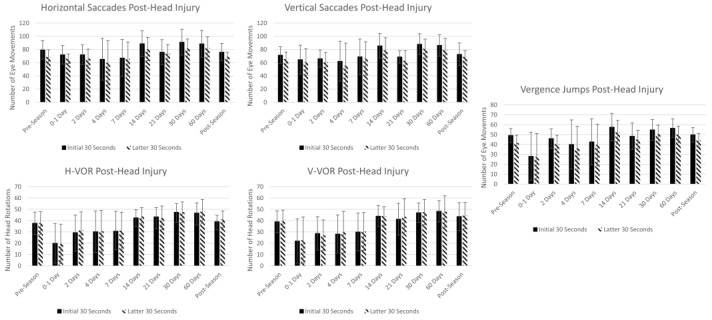
Average for all 5 timed assessments in players at pre-season and post-head injury.

**Figure 4:**
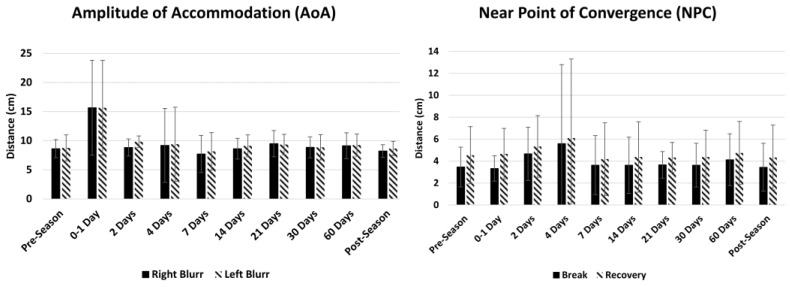
Average of the 2 non-timed assessments (amplitude of accommodation [AoA] and near point of convergence [NPC]) in players at pre-season and post-head injury.

The average with one standard deviation for the initial and latter 30 s for each of the five assessments in 15 athletes is demonstrated in Figure 3 and Table 3. The linear mixed model showed statistical differences in the time point fixed effect for all the measurements (*p* < 0.001); however, the endurance (initial vs. latter 30 s) fixed effect was only statistically significant in horizontal saccades (*p* = 0.034) and vergence jumps (*p* = 0.018). A trending decrease was observed in the five timed tests; within the first 0–4 days post-head injury, there were decreases in the number of eye movements, which increased 14–60 days post-head injury. This trend is the most robust in the vergence jumps, H-VOR, and V-VOR tests. There is a trending increase in AoA immediately following a head injury and a trending increase in NPC by day 4 in the Division I athletes, as demonstrated in Figure 4.

## 4. Discussion

This study was conducted to establish normative data and determine the repeatability and reliability of the OculoMotor & Vestibular Endurance Screening (MoVES) on Division I athletes during the pre-season, post-season, and post-head injury. While vestibular/ocular motor screening (VOMS) has been shown to distinguish between non-concussed and concussed by quantifying symptom provocation upon the application of vestibular/ocular tests, [8] symptom reporting is subjective, which may inherently affect the results [20,21]. The MoVES assessment not only captures reported symptoms following 10 repetitions but also captures the total number of eye movements that can be made within 1 min. As compared to the limited 10 repetitions captured by the VOMS, the MoVES assessment captures the total number of eye movements that can be performed within one minute. This allows the clinician/researcher to assess endurance by comparing the number of observed eye movements in the initial 30 s and the latter 30 s [19,22,23,24,25]. Additionally, the MoVES assessment not only records near point of convergence (NPC) but also has a screening test to quantify the dynamics of vergence, which has been reported to be critically affected in concussed athletes [26,27,28,29]. Finally, the majority of concussion screenings avoid measuring the amplitude of accommodation, which has been included in the MoVES. 

Normative and repeatability data were collected in Division I athletes at pre-season and post-season and in a subset of Division I players who experienced a head injury. Data were collected on 0–1, 2, 4, 7, 14, 21, 30, and 60 days post-head injury. We proposed the following two hypotheses: (1) there would be trending decreases in the number of observed eye or head movements immediately post-head injury that resolve 2–4 weeks post-head injury, and (2) there would be decreases in the total number of eye or head movements that persist beyond 4 weeks, which may indicate persistent oculomotor and vestibular dysfunctions requiring therapeutic intervention for resolution. The findings herein provide initial evidence for the use of MoVES to identify athletes with an oculomotor and/or vestibular problem following sports-related head injury. The MoVES assessment may enhance current concussion assessments to comprehensively evaluate outcomes in patients with vestibular and/or ocular deficits, leading to more targeted interventions.

### 4.1. Horizontal and Vertical Saccades

Video oculography studies have found that saccadic eye movements show variable amplitude and velocity following a sports-related concussion [21]. Prior studies also confirm there is a decrease in velocity as the number of saccades increases due to fatigue in individuals with binocular vision [30,31]. Although this cohort was composed of Division I athletes, similar to our prior study, there was a decrease in vertical saccades and horizontal saccades in the latter 30 s [19] both during pre-season and post-season. Even though there was a statistically significant difference between horizontal saccades in the initial 30 s pre-season versus post-season, as well as horizontal and vertical saccades in the latter 30 s pre-season versus post-season, we concluded that this outcome is not clinically meaningful because the mean difference between the groups was less than one standard deviation. It is normal to expect, on average, a decrease of two to seven saccadic movements in healthy participants. However, it is expected that in athletes with a concussion, the number of observed eye movements in the latter 30 s will be even lower than in the latter 30 s of the control participants [22].

#### 4.1.1. Vergence Jumps

The VOMS examines the dynamics of saccades, but not the vergence system, which is critically affected post-concussion. The incidence of vergence deficits post-concussion is greater (38–49%) in children [3,32] and adults [33,34] compared to non-concussed individuals. As a result, it is essential to understand clinical measures and eye movement characteristics for vergence [24]. Studies in binocularly normal controls show decreases of up to 20% in vergence peak velocity as the recording of convergence eye movements progresses over time [22,35]. This study confirms that the number of observed vergence jumps decreases in the latter 30 s compared to the initial 30 s. This study also shows that there was no difference between the initial 30 s at pre-season compared to the initial 30 s post-season or between the latter 30 s at pre-season compared to the latter 30 s post-season. Thus, decreases in vergence performance may be able to differentiate between normal controls and concussed individuals. The MoVES assessment’s measurement of vergence eye movements serves as an indirect measure and representation of peak velocity which has been shown to decrease post-concussion [22,36,37,38].

#### 4.1.2. Vertical and Horizontal VOR

Prior studies have reported that vestibular impairments are commonly reported post-concussion and may lead to delayed recovery [39,40]. The vestibular system is related to the visual system through the vestibular-ocular reflex (VOR) [41]. Current clinical tools, such as the Balance Error Scoring System and King–Devick, examine vestibular, balance, and visual ocular systems post-concussion [42,43,44]. The VOMS attempts to test a participant’s VOR and ability to stabilize vision by only assessing symptoms evoked during 10 repetitions of vertical and horizontal head and eye movements [8]. This study applied MoVES assessment, which observed the number of vertical and horizontal rotations for 1 min and found that there were no differences in V-VOR and H-VOR between pre-season and post-season outcomes in the initial 30 s or latter 30 s. This screening measures a person’s VOR in the initial 30 s and latter 30 s, allowing clinicians to indirectly examine post-concussive vestibular endurance that cannot be objectively captured by existing assessments. 

#### 4.1.3. Amplitude of Accommodation (AoA) and Near Point of Convergence (NPC) 

Concussions are known to impair the visual pathways involved in accommodation, vergence, and visual tracking [34,45,46]. In fact, near point of convergence (NPC) and accommodative amplitude (AA) have been shown to be the best predictors of post-concussion symptoms [3]. Therefore, the MoVES assessment included both tests. This study demonstrated that there were no significant differences in NPC or AA at pre-season compared to post-season. The normal range of AA is based on a person’s age, so this population should have values below 10 cm, which was observed in pre-season and post-season. The normal range of NPC break was less than 6 cm from the nasion, which is observed at both time points.

#### 4.1.4. Trends in Athletes at Pre-Season, Post-Head Injury, and Post-Season

Some of the injured athletes were unable to perform the tests during each of the time points because they were advised by medical personnel to limit screen time after a suspected head injury, which made it difficult to schedule data collection for this subset. This resulted in some players being unavailable at each of the time points (0–1, 2, 4, 7, 14, 21, 30, and 60 days), leading to small sample sizes and making it challenging to accurately interpret statistical tests. The initial results show that slight decreases were observed in the number of eye movements during vertical and horizontal saccades in the initial days post-head injury, but these changes are not robust. Deficits in saccadic eye movements have previously been observed post-concussion; in fact, studies have found that fixational saccade amplitude is greater and faster post-concussion [21,47,48]. Although this study needs more participants to confirm there is not a clinically significant change between pre-season and post-injury outcomes in the number of saccadic eye movements, a recent study has also demonstrated that there is no difference between the number of saccades completed in concussed individuals compared to controls [49].

Intriguingly, trending decreases in the number of eye movements were seen acutely (0–1 day) post-head injury in H-VOR, V-VOR, and vergence jumps. While the VOMS assessment evaluates symptoms following 10 repetitions of the H-VOR and 10 repetitions of the V-VOR [8], the nature of the test does not evaluate the number of eye movements the patient is able to complete within a certain time, nor vergence jumps. The performance of 10 repetitions may not be sufficient to evoke symptoms in all the participants who have experienced a head injury. The MoVES assessment may be able to use the number of eye movements completed in each of its timed tests to detect subtle changes that may distinguish healthy controls from athletes with vestibular and/or ocular dysfunction post-concussion. A larger number of movements or a longer test is required to observe the effects of endurance. Additionally, there was also a trending increase at 0–1 days in AoA and an increase in NPC on day 4. These initial trends align with studies that show deficits in AoA and NPC in patients’ post-concussion [32,50]. Lastly, for all tests, there is a trending increase suggesting improvement near pre-season measures by day 14. These initial findings align with studies that report 80 percent of concussions resolve over 7 to 14 days [51,52,53].

## 5. Limitations

There are some limitations to this study. The participants were adolescent Division I athletes, so it is unclear if these outcomes are representative of the general population. In fact, the visual and vestibular systems change with aging, so future studies should investigate differences between younger and older populations [54,55,56]. Additionally, an optimal method to measure endurance may involve shortening the bins to 15 s to obtain an understanding of endurance post-concussion [22]. Future segregation of data into smaller bins (15 or 10 s bins) may allow the assessment to be a total of 30 s each instead of a full minute. Additionally, the athletes in this study were not diagnosed by a physician. To validate this tool, a more robust data set of diagnosed concussion patients is needed. The MoVES assessment can be considered effective as long as a decrease in the number of eye movements or head rotations can be observed in a participant with a head injury. Finally, the 15 participants in the head injury group with varying time points are not sufficient to make any broad statements. This study shows early feasibility, and a larger study must be conducted to observe the trends in injured participants.

## 6. Conclusions

The MoVES assessment may use the patient’s ability to perform a specific number of eye movements or head rotations, denoted as endurance, to determine vestibular/oculomotor recovery in patients’ post-concussion. This study demonstrates initial data on the use of MoVES assessment as a portable screening tool that may be used to detect vestibular and oculomotor impairments post-head injury or impact. The quantitative nature of the assessment instrument could also make it an asset for determining “return-to-play” or “return-to-work” protocols. With further research, MoVES could be utilized as a standalone oculomotor and vestibular assessment without the need for pre-season measurements. 

## Figures and Tables

**Table 1:** Descriptive statistics for all seven assessments in the normative cohort (*n* = 311) collected at pre-season.

Test	Time Interval	Average (±SD)	Non- Zero Minimum	Maximum	Median (95% Confidence Interval)
Horizontal Saccades	Initial 30	73.5 ± 13.3	38	140	74 (72–75)
	Latter 30	66.6 ± 11.6	34	102	66 (65–68)
	Total	140.0 ± 24.1	72	242	140 (137–143)
Vertical Saccades	Initial 30	69.7 ± 11.8	40	104	68 (68–71)
	Latter 30	65.2 ± 11.3	22	88	66 (64–66)
	Total	134.9 ± 22.3	68	188	136 (132–137)
Vergence Jumps	Initial 30	45.7 ± 11.9	6	86	44 (44–47)
	Latter 30	43.0 ± 11.7	5	90	42 (42–44)
	Total	88.7 ± 22.9	10	172	86 (86–91)
Horizontal VOR	Initial 30	36.6 ± 11.1	6	84	36 (35–38)
	Latter 30	36.7 ± 10.6	2	74	36 (36–38)
	Total	73.3 ± 21.2	12	158	72 (71–76)
Vertical VOR	Initial 30	37.3± 10.9	8	74	36 (36–38)
	Latter 30	38.0 ± 10.8	10	68	38 (37–39)
	Total	75.3 ± 21.3	20	140	74 (73–78)
Near Point of Convergence	Break (cm)	3.3 ± 2	2	10	3 (3–4)
	Recovery (cm)	4.4 ± 2	2	16	4 (4–5)
Amplitude of Accommodation	Right (cm)	8.3 ± 2	4.5	16	8 (8–9)
	Left (cm)	8.6 ± 2	4.0	16	8 (8–9)

**Table 2:** Descriptive statistics for all seven assessments for players (*n* = 120) with pre-season and post-season data.

		Pre-Season				Post-Season			
Test	Time Interval	Average (±SD)	Non- Zero Minimum	Maximum	Median (95% Confidence Interval)	Average (±SD)	Non- Zero Minimum	Maximum	Median (95% Confidence Interval)
Horizontal Saccades	Initial 30	74.1 ± 12	46	102	74 (72–76)	76.8 ± 14	40	116	76 (74–79)
	Latter 30	67.1 ± 11	38	94	68 (65–69)	70.5 ± 13	38	112	70 (68–72)
	Total	141.2 ± 23	90	194	142 (137–145)	147.3 ± 27	78	224	144 (142–152)
Vertical Saccades	Initial 30	70.3 ± 11	44	96	69 (68–72)	72.1 ± 13	38	104	72 (70–74)
	Latter 30	65.6 ± 10	36	88	66 (63–67)	68.5 ± 13	38	106	68 (66–71)
	Total	135.9 ± 21	86	184	136 (132–140)	140.6 ± 25	78	206	140 (136–145)
Vergence Jumps	Initial 30	48.4 ± 12	20	86	48 (46–51)	49.9 ± 11	28	82	49 (48–52)
	Latter 30	45.7 ± 13	24	90	46 (43–48)	46.7 ± 10	24	76	44 (45–49)
	Total	94.1 ± 24	40	172	96 (90–98)	96.6 ± 21	54	154	94 (93–100)
Horizontal VOR	Initial 30	38.2 ± 11	14	74	38 (36–40)	36.3 ± 10	12	74	36 (34–38)
	Latter 30	37.6 ± 11	2	68	36 (36–39)	37.1 ± 10	12	74	36 (35–39)
	Total	75.7 ± 21	32	134	76 (72–80)	73.4 ± 20	26	148	72 (70–77)
Vertical VOR	Initial 30	38.2 ± 11	20	66	36 (36–40)	37.1 ± 11	18	80	35 (35–39)
	Latter 30	37.6 ± 11	2	68	36 (36–40)	38.2 ± 11	18	80	36 (36–40)
	Total	76.2 ± 21	36	134	73 (72–80)	75.4 ± 21	36	156	71 (71–79)
Near Point of Convergence	Break (cm)	3.7 ± 2	2	9.5	3 (3–4)	3.5 ± 2	2	9.5	3 (3–4)
	Recovery (cm)	4.9 ± 2	2	12.5	5 (4–5)	5.0 ± 2	2	11.5	5 (4–5)
Amplitude of Accommodation	Right (cm)	8.3 ± 2	4.5	14.0	8 (8–9)	8.5 ± 2	4.5	16.0	9 (8–9)
	Left (cm)	8.5 ± 2	4.5	15.5	9 (8–9)	8.5 ± 2	4.5	16.0	9 (8–9)

**Table 3:** Longitudinal MoVES data on athletes who experienced a head injury.

Time Point	Horizontal Saccades		Vertical Saccades		Vergence Jumps		Horizontal VOR		Vertical VOR		Amplitude of Accommodation		Near Point of Convergence	
	Initial 30 s	Latter 30 s	Initial 30 s	Latter 30 s	Initial 30 s	Latter 30 s	Initial 30 s	Latter 30 s	Initial 30 s	Latter 30 s	Right (cm)	Left (cm)	Break (cm)	Recovery (cm)
Pre-Season (*n* = 15)	79.4 ± 14	68.8 ± 11	71.5 ± 13	65.4 ± 11	49.1 ± 7	41.4 ± 8	37.7 ± 10	37.8 ± 10	39.1 ± 10	39.5 ± 10	8.6 ± 2	8.8 ± 2	3.5 ± 2	4.5 ± 3
0–1 Day (*n* = 3)	72.0 ± 14	66.0 ± 7	64.7 ± 22	60.7 ± 20	28.0 ± 24	27.3 ± 24	20.0 ± 17	19.3 ± 17	22.0 ± 20	22.7 ± 21	15.7 ± 8	15.7 ± 8	3.3 ± 1	4.7 ± 2
2 Days (*n* = 7)	72.0 ± 15	66.0 ± 15	66.0 ± 13	61.0 ± 14	46.0 ± 10	40.3 ± 9	29.3 ± 16	31.3 ± 16	28.7 ± 15	27.0 ± 14	8.8 ± 2	9.8 ± 1	4.7 ± 2	5.3 ± 3
4 Days (*n* = 11)	65.2 ± 32	59.6 ± 33	62.2 ± 30	55.6 ± 34	40.0 ± 25	36.0 ± 22	30.2 ± 18	30.6 ± 18	28.2 ± 17	29.8 ± 18	9.2 ± 6	9.4 ± 6	5.6 ± 7	6.1 ± 7
7 Days (*n* = 12)	67.1 ± 28	65.6 ± 26	68.9 ± 27	66.0 ± 26	42.7 ± 23	39.5 ± 21	30.7 ± 17	31.1 ± 16	30.0 ± 17	30.4 ± 17	7.7 ± 3	8.2 ± 3	3.6 ± 3	4.2 ± 3
14 Days (*n* = 9)	88.8 ± 20	80.5 ± 18	85.5 ± 19	78.8 ± 19	57.5 ± 14	52.3 ± 12	42.5 ± 7	43.7 ± 8	44.0 ± 9	44.0 ± 8	8.6 ± 2	9.1 ± 2	3.6 ± 3	4.4 ± 3
21 Days (*n* = 7)	76.0 ± 19	74.0 ± 13	68.7 ± 9	63.0 ± 15	48.3 ± 13	45.0 ± 10	43.3 ± 8	42.3 ± 11	41.3 ± 14	43.3 ± 16	9.5 ± 2	9.4 ± 2	3.7 ± 1	4.3 ± 1
30 Days (*n* = 9)	91.0 ± 20	81.5 ± 14	88.0 ± 16	81.3 ± 15	54.8 ± 10	50.5 ± 9	47.5 ± 8	47.5 ± 9	47.0 ± 9	47.5 ± 11	8.9 ± 2	8.9 ± 2	3.6 ± 2	4.4 ± 2
60 Days (*n* = 9)	88.5 ± 20	82.0 ± 17	86.3 ± 16	79.0 ± 18	56.5 ± 9	50.0 ± 8	46.8 ± 9	48.0 ± 11	48.3 ± 9	48.0 ± 14	9.1 ± 2	9.3 ± 2	4.1 ± 2	4.7 ± 3
Post-Season (*n* = 15)	76.0 ± 13	68.9 ± 7	72.7 ± 17	68.7 ± 9	49.8 ± 7	44.2 ± 7	39.1 ± 6	41.1 ± 8	43.6 ± 12	44.4 ± 12	8.2 ± 1	8.7 ± 1	3.4 ± 2	4.3 ± 3

## Data Availability

The data that support the findings of this study are available from the corresponding author, C.Y., upon reasonable request. The data are not publicly available due to privacy and ethical restrictions.

## Supplementary Materials

The following supporting information can be downloaded at https://www.mdpi.com/article/10.3390/brainsci14070704/s1: Supplementary S1: MoVES Protocol (https://research.njit.edu/sqrl/moves (accessed on 28 May 2024)); Video S1: A demonstration of horizontal and vertical VOR movements to be made by the participants.
