# Supplementary material for: OculoMotor & Vestibular Endurance Screening (MoVES) Normative, Repeatability, and Reliability Data
Source: Brain Sci. 2024 Jul 14;14(7):704. doi: 10.3390/brainsci14070704 (PMC11274463; doi:10.3390/brainsci14070704)
Supplement: Supplementary file 1 [file brainsci-14-00704-s001.zip › AppendixA_V2.pdf]

OculoMotor & Vestibular Endurance Screening (MoVES)

Screening	1st 30 seconds	2nd 30 seconds	Headache 0 – 10	Dizziness 0 – 10	Nausea 0 – 10	Fogginess 0 – 10	Comments
Baseline							
OculoMotor Assessment							
Horizontal Saccades							
Vertical Saccades							
Near Point of Convergence	Break (cm): ___________	Recovery (cm): _____					
Amplitude of Accommodation	Right Blur (cm): _____	Left Blur (cm): _____					
Vergence Jumps							
Vestibular/OculoMotor Reflex (VOR)							
Horizontal VOR							
Vertical VOR							

Instructions

All tests, except for accommodative amplitude, should be performed with participants wearing their corrective refraction (glasses or contacts). The screening should be stopped if the patient reports any discomfort or symptoms including headache, dizziness, nausea, fogginess, light-headedness, or loss of balance. After each test, the examiner should ask the patient to report their symptoms in terms of headache, dizziness, nausea, and fogginess on a scale of 0 to 10, where 0 is no symptoms and 10 is severe symptoms.

Tools

This screening is best performed by utilizing the OculoMotor Assessment Tool (OMAT) and its companion OMAT smartphone app. The OMAT has four (4) components: 1) Vergence Bar, 2) Saccade Bar, 3) Large Slider, and 4) Small Slider.

OculoMotor Screening:

Saccadic Eye Movements:

Screening Procedure for Horizontal Saccades: This screening measures a person's ability to rapidly move their eyes in the horizontal plane. The examiner should attach the Saccade Bar to the Vergence bar via the magnet. *Instruct the patient to move their eye rapidly between the left and right "X" targets. Inform the patient that it is important to not overshoot or undershoot the target "X"'s.* The examiner should place the 1cm end of the vergence bar (with the saccade bar attached at the 25cm end) on the patient's nasion (Figure 2). The two (2) "X" targets should be facing the patient with the green "X" on the patient's left side and the red "X" on the patient's right side. The examiner should ensure that the saccade bar is parallel to the ground and at the patient's eye level. The examiner should select the "HORIZONTAL" option in the OMAT smartphone application. *Instruct the patient to start on the green "X". When both the examiner and patient are ready, instruct the patient to begin the task.* The counter and timer on the app will begin when the "COUNT" or "START" button is pressed on the OMAT smartphone application. The large "COUNT" button should be pressed for every eye movement. Left to right is one movement, right to left is another movement. *Instruct the patient to stop when 60 seconds have elapsed.* The OMAT smartphone application will make a sound and vibrate when 60 seconds have elapsed.

Screening Procedure for Vertical Saccades: This screening measures a person's ability to rapidly move their eyes in the vertical direction. The instructions for this procedure are the same as the ones listed for *Horizontal Saccades* with one exception. Rotate the OMAT tool with the Saccade Bar attached to the Vergence Bar 90 degrees, so that the both target "X"'s are facing the patient and the green "X" is above the patient's eye line and the red "X" is below the patient's eye line. *Instruct the patient to move their eye rapidly between the two top and bottom "X" targets. Inform the patient that it is important to not overshoot or undershoot the target "X"'s.* The examiner should also select the "VERTICAL" option in the OMAT smartphone application. *Instruct the patient to the start on the green "X" (top). When both the examiner and patient are ready, instruct the patient to begin the task.* The counter and timer on the app will begin when the "COUNT" or "START" button is pressed on the OMAT smartphone application. The large "COUNT" button should be pressed for every eye movement. Top to bottom is one movement, bottom to top is another movement. *Instruct the patient to stop when 60 seconds have elapsed.* The OMAT smartphone application will make a sound and vibrate when 60 seconds have elapsed.

Near Point of Convergence:

Screening Procedure for NPC: This screening measures a person's ability to converge (cross) their eyes as a target approaches them. The examiner should first remove the Saccade Bar from the Vergence Bar, if still installed from Saccadic Eye Movement screening. The examiner should then insert the Large Slider into the Vergence Bar, ensuring that the letters of the slider are facing towards the 1cm marking. Position the slider furthest away from the patient at the 25cm marking. *Instruct the patient to maintain fixation on the letters, making sure that the letters are single. The patient should report when the letters become double and when they become single again.* Some individuals may perceive the letters as blurry, but blurred vision is not the endpoint for this test. The examiner should place the 1cm end of the vergence bar on the patient's nasion and slowly (~ 2 cm/second) move the slider towards the patient's nose. The examiner should remind the patient to fixate on the letters, maintaining single vision. When the patient reports doubling, the examiner should ask the patient to attempt to fuse the letters and make them single again. If the patient is able to fuse the letters to be single, continue moving the slider closer to the patient's eyes. ***Record the cm value (to the nearest ½ centimeter) where the patient reports sustained double vision.*** When the patient can no longer fuse the letters, move the slider away from the patient, and ***record the centimeter value where the patient perceives the letters as single again***.

> **Normative Data:** Normal range of near point of convergence doubling is less than 6cm from the nasion.

Amplitude of Accommodation:

Screening Procedure for Accommodative Amplitude: This screening measures a person's optical power of each eye. The instructions for this procedure are similar to the ones listed for *Near Point of Convergence*, except it is important to make sure that the patient is not using reading glasses or a bifocal/progressive lens. If the patient wear bifocals or progressive lenses, make sure they view the target through the top of the glasses for this test. Instead of placing the OMAT (with the Vergence bar and Large slider) on the nasion, the 1cm end of the Vergence bar should first be placed above the right eyebrow (Figure 2). *Instruct the patient to cover their left eye with their left hand and maintain focus on the letters, making sure that the letters are clear. The patient should report when the letters become blurry.* The examiner should place the 1cm end of the vergence bar on top of the patient's right eyebrow and slowly (~ 2cm/second) move the slider towards the patients nose. The examiner should remind the patient to focus on the letters, maintaining them clear. ***Record the cm value (to the nearest centimeter) of when the letters appear blurry for the patient.***

Repeat the procedure for the left eye, with the vergence bar above the left eyebrow, and the patient covering their right eye with their right hand.

> **Normative Data:** Normal range of accommodative amplitude is calculated based on the patient's age. Refer to Table 1 for normalcy values; values below the line are normal. For screening purposes use 15-1/4 age-2D

Table 1: Amplitude of Accommodation normal range based on age

Age (years)	Monocular Amplitude of Accommodation (Diopters)
6-8	13
9-12	12
13-16	11
17-20	10
21-24	9
25-28	8
29-32	7
33-36	6
37-40	5
41-44	4

Vergence Eye Movements:

Screening Procedure for Vergence Jumps: This screening measures a person's ability to rapidly converge and diverge their eyes as they fixate a far and near target. The examiner should insert both the Large and Small sliders into the Vergence Bar, ensuring that the solid lines on the sliders are facing towards the 1cm marking. The Large slider should be placed at position "A" (~25 cm) and the Small slider should be placed at position "F" (~9 cm). *Instruct the patient to rapidly change their fixation from the far (Larger slider) to near (Small slider) target. The patient should change fixation from one target to the other until they achieve single vision.* The examiner should select the "VERGENCE" option in the OMAT smartphone application. The examiner should place the 1cm end of the vergence bar on the patient's nasion. *Instruct the patient to the start on the further target (Larger slider). When both the examiner and patient are ready, instruct the patient to begin the task.* The counter and timer on the app will begin when the "COUNT" or "START" button is pressed on the OMAT smartphone application. The larger "COUNT" button should be pressed for every eye movement. Every time the patient converges their eyes is one movement, and every time the patient diverges their eyes is another movement. *Instruct the patient to stop when 60 seconds have elapsed.* The OMAT smartphone application will make a sound and vibrate when 60 seconds have elapsed.

Vestibular Screening:

Horizontal Vestibular-OculoMotor Reflex:

Screening Procedure for Horizontal VOR: This screening measures a person's vestibular (balance/stabilization) function by maintaining their fixation on a target as they move their head horizontally. Only the Larger slider is required for this screening. *Instruct the patient to completely extend their non-dominant hand holding the Larger slider at their eye level between their eyes with the letters facing the patient. The patient should rotate their head horizontally in the left and right directions as far and as fast as they can while maintaining fixation on the letters with both their eyes.* The examiner should select the "HORIZONTAL" option in the OMAT smartphone application. *Instruct the patient to start with their face turned to the left direction while maintaining fixation on the letter. When both the examiner and patient are ready, instruct the patient to begin the task.* The counter and timer on the app will begin when the "COUNT" or "START" button is pressed on the OMAT smartphone application. The larger "COUNT" button should be pressed for every full head motion. Every time the patient turns their head from left to the right is one movement, and every time the patient turns their head right to left is another movement. *Instruct the patient to stop when 60 seconds have elapsed.* The OMAT smartphone application will make a sound and vibrate when 60 seconds have elapsed.

Screening Procedure for Vertical VOR: This screening measures a person's balance by maintaining their fixation on a target as they move their head vertically. The instructions for this procedure are the same as the ones listed for *Horizontal VOR* with few exceptions. The patient should rotate their head up and down while holding the Large slider with their dominant hand or different hand from the one used in Horizontal VOR, this is to avoid arm fatigue. The examiner should also select the "VERTICAL" option in the OMAT smartphone application and press the "COUNT" or "START" button when they are ready to start counting the number of eye movements. Every time the patient turns their head from up to the down is one movement, and every time the patient turns their head down to up is another movement. *Instruct the patient to stop when 60 seconds have elapsed.* The OMAT smartphone application will make a sound and vibrate when 60 seconds have elapsed.

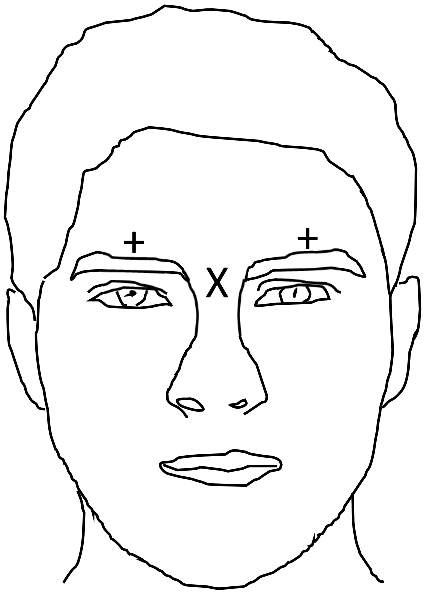

Figure 1: Location “X” is the nasion. The vergence bar should be placed at this location for all the oculomotor screening expect accommodative amplitude. Location “+” is the location where the vergence bar should be placed for accommodative amplitude for each eye.

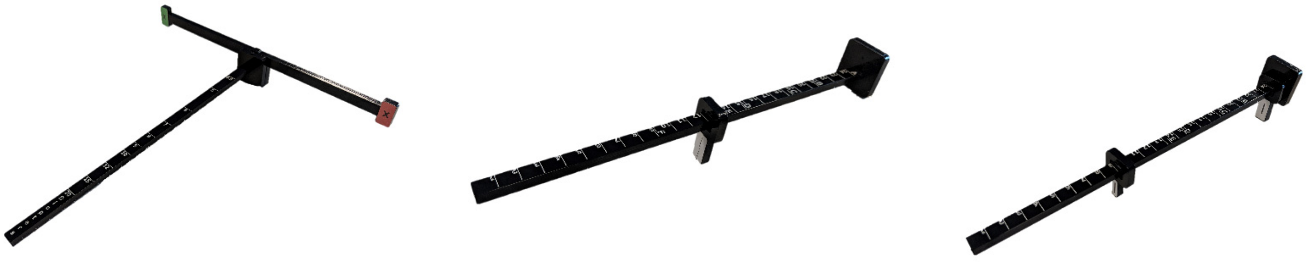

Figure 2: OMAT Configuration for Saccadic Testing (Left), Near Point of Convergence/ Amplitude of Accommodation (Middle), and Vergence Jumps (Right)
